# Clinical applications of antibody-drug conjugates in advanced non-small cell lung cancer

**DOI:** 10.3389/fimmu.2026.1787148

**Published:** 2026-02-18

**Authors:** Hai Guo, Zhongcai Xu, Kaidi Li, Chenglin Guo, Feng Lin, Qiang Pu, Guosong Wang

**Affiliations:** 1 Department of Thoracic Surgery, West China Hospital, Sichuan University, Chengdu, Sichuan, China; 2 Department of Thoracic Surgery, Yuxi Traditional Chinese Medicine Hospital, Yuxi, Yunnan, China; 3 Department of Experimental Research, Sichuan Clinical Research Center for Cancer, Sichuan Cancer Hospital & Institute, Sichuan Cancer Center, University of Electronic Science and Technology of China, Chengdu, China

**Keywords:** antibody–drug conjugates, cancer, clinical application, NSCLC, targeted therapy

## Abstract

Lung cancer remains the leading cause of cancer-related incidence and mortality worldwide, with non-small cell lung cancer (NSCLC) constituting the majority of cases. Current treatment modalities are constrained by significant limitations: conventional chemotherapy exhibits poor tumor selectivity and systemic toxicity, while monoclonal antibodies frequently demonstrate inadequate therapeutic efficacy. Antibody-drug conjugates (ADCs)—engineered biotherapeutics comprising tumor-targeting antibodies conjugated to potent cytotoxic agents through optimized linkers—have emerged as a transformative strategy to address these therapeutic challenges in advanced NSCLC. This review systematically examines the structural architecture, developmental evolution, and mechanistic foundations of ADCs, with a focused evaluation of clinical evidence supporting ADCs targeting HER2, TROP2, c-MET, HER3, CEACAM5, and B7−H3 in advanced NSCLC. We critically assess efficacy outcomes, safety profiles, predictive biomarkers, and toxicity management strategies—particularly regarding interstitial lung disease, hematologic toxicities, and neuropathic adverse events. Key clinical challenges including tumor heterogeneity, therapeutic resistance, biomarker heterogeneity, and patient stratification are analyzed. Furthermore, we highlight emerging therapeutic approaches such as next−generation ADC design, novel linker-payload systems, bispecific platforms, and rational combination strategies with targeted and immunotherapeutic agents. Collectively, these developments position ADCs as promising precision oncology tools capable of reshaping treatment paradigms and improving clinical outcomes in advanced NSCLC.

## 1 Introduction

Cancer is the second leading cause of mortality worldwide. There were 18.5 million incident cases of cancer and 10.4 million cancer-related mortalities globally in 2023. Every 3 seconds, a new cancer case was diagnosed, and every 6 seconds, a cancer-related mortality occurred. Lung cancer remains the dominant contributor to cancer-related mortality, with an estimated 2.48 million new cases and 1.82 million deaths in 2022 (ASIR 23.6/100, 000; ASMR 16.8/100, 000) (1). Lung cancer is broadly classified into small cell lung cancer (SCLC) and non-small cell lung cancer (NSCLC). NSCLC is the most prevalent histological subtype, accounting for approximately 85% of all diagnosed lung cancers (2). Consequently, the threat posed by NSCLC to human health cannot be overlooked.

Although patients with early-stage NSCLC generally have a favorable prognosis, a significant proportion are initially diagnosed with locally advanced disease (Stage IIIB-IV) due to the aggressive characteristics of NSCLC, such as rapid proliferation and high potential for metastasis (3). Surgical treatment plays a role for resectable advanced NSCLC (4); however, its inability to effectively stimulate anti-tumor immunity often fails to prevent cancer recurrence and metastasis. For unresectable cases, multimodal therapies, including radiotherapy, chemotherapy, targeted therapy, and immunotherapy, have become established first-line treatment options (5, 6). Despite advances in targeted therapy and immune checkpoint inhibitors, the five-year survival rate for advanced NSCLC remains around 15–20%. Factors such as treatment resistance, brain metastasis, and drug-related toxicities (e.g., severe neutropenia, organ toxicities, and generalized debilitation) are ultimately associated with diminished overall survival (OS) (7–9). Therefore, there is a pressing need for more effective and less toxic novel agents for NSCLC treatment.

Antibody-drug conjugates (ADCs) have emerged as a key strategy to overcome drug resistance and expand the treatment landscape for advanced NSCLC (10–12). ADCs are a class of targeted anticancer therapeutics composed of monoclonal antibodies (mAbs), highly potent cytotoxic payload and specialized linkers (13). ADCs leverage the precision of monoclonal antibody targeting to deliver cytotoxic molecules specifically to tumor cells, achieving highly efficient tumor cell killing while demonstrating potential for synergistic effects with immunotherapy. Unlike the non-specifical nature of conventional chemotherapy, ADCs achieve targeted release of cytotoxic payloads via antibody-mediated delivery, thereby significantly reducing systemic toxicity. Furthermore, the bystander effect may potentially contribute to antitumor activity in antigen-negative, heterogeneous or prior therapy-resistant tumors. Additionally, ADCs can trigger immunogenic cell death, potentially augmenting antitumor immune responses (14–16).

Since the initial regulatory approval of the first agent in 2000, the development of ADCs has progressed through more than two decades of continuous innovation. Gemtuzumab Ozogamicin became the first FDA-approved ADC for CD33-positive acute myeloid leukemia, marking a significant milestone for first-generation ADCs in clinical practice (17, 18). Subsequently, second-generation ADCs (such as Brentuximab Vedotin and ado-Trastuzumab Emtansine) emerged with improved linker stability and controlled drug-to-antibody ratio (DAR), representing a major breakthrough in solid tumor treatment (19, 20). Building upon these advances, third-generation ADCs (such as Trastuzumab Deruxtecan, Sacituzumab Govitecan) are characterized by optimized site-specific conjugation, antibody engineering, linker design, and payload selection, resulting in enhanced therapeutic efficacy and an improved safety profile, thereby establishing ADCs as crucial components in systemic cancer therapy (21, 22).

To date, ADCs have been approved for various malignancies, including breast cancer, hematologic cancers, gynecologic cancers, and advanced NSCLC (23, 24). This review systematically examines the mechanisms of action, targetable antigens, and corresponding biomarker profiles of ADCs in advanced NSCLC. By synthesizing critical clinical evidence on therapeutic efficacy, safety profiles, and patient selection strategies, along with practical management approaches for ADC-associated toxicities, this work bridges the gap between fundamental research and clinical practice. Ultimately, it provides valuable guidance for the personalized and precise treatment of NSCLC, enhancing both the efficacy and safety of ADCs in the management of advanced NSCLC.

## 2 Composition, evolution, and mechanism of action of ADCs

The conceptual foundation for ADCs traces back to Paul Ehrlich’s visionary “magic bullet” hypothesis, which proposed the development of therapeutics capable of selectively targeting diseased tissues while sparing healthy cells (13). Building this theory, ADCs are engineered to deliver highly potent cytotoxic agents specifically to tumor cells by linking them via optimized linkers to mAbs that recognize tumor-associated antigens. This design enables high intratumoral drug concentrations while minimizing systemic exposure (14, 25, 26).

The mAbs utilized in ADCs predominantly consist of humanized IgG1 or IgG4 subtypes, which reduce immunogenicity and prolong plasma half-life (25, 27). These mAbs employed in ADCs confer precise target recognition through specific binding to surface antigens on malignant cells. In NSCLC, clinically validated and emerging molecular targets include receptor tyrosine kinases (e.g., human epidermal growth factor receptor 2(HER2), human epidermal growth factor receptor 3(HER3), epidermal growth factor receptor(EGFR), and mesenchymal–epithelial transition (c-MET)), tumor-associated antigens (e.g., trophoblast cell surface antigen 2 (TROP2), carcinoembryonic antigen–related cell adhesion molecule 5 (CEACAM5), B7 Homolog 3(B7-H3), folate receptor alpha(FRα), tissue factor(TF), mucin 1(MUC1)) and cell adhesion molecules (e.g., nectin cell adhesion molecule 4 (NECTIN4)) (**Table 1**) (30–41, 43–50).

**Table 1:** Protein/gene targets in lung cancer: expression and prognosis.

Target	Description (Biological function)	Expression rate in lung cancer (%)	Prognostic association
HER2 (28, 29)	RTK in HER family; drives MAPK/PI3K for proliferation and anti-apoptosis (ERBB2 gene)	10-20 (overexpression/amplification)	Poor (high recurrence, short OS)
HER3 (28–31)	RTK in HER family; dimerization partner activating PI3K/AKT for proliferation and survival	50-70 (high in EGFR-mutated NSCLC)	Poor (shorter PFS/OS, invasion)
TROP-2 (32–34)	Transmembrane glycoprotein; promotes EMT and stemness (TACSTD2 gene)	80 (high in adenocarcinoma)	Poor (invasion, metastasis, short PFS/OS)
MET (35–39)	RTK activated by HGF; invasion and angiogenesis (c-MET gene)	3-5 (mutations), 15-20 (high expression)	Poor (TKI resistance, metastasis)
B7-H3 (40, 41)	Immune checkpoint; inhibits T-cell activation (CD276 gene)	70 (high in SCLC/NSCLC)	Poor (immune escape, short OS)
PD-L1/B7-H4 (40)	Immune checkpoints; negative T-cell regulation (CD274/VTCN1 genes)	20-30 (PD-L1), ~50 (B7-H4) in SCLC	Poor (immune suppression, short OS)
EGFR (38, 39, 42)	RTK driving proliferation; mutations activate signaling (EGFR gene)	10-40 (mutations), widespread overexpression	Good initial response.Poor in resistance)
FRα (43)	Transmembrane glycoprotein; folate uptake for DNA synthesis (FOLR1 gene)	30-40 (high in adenocarcinoma)	Poor (recurrence risk)
TF (44)	Transmembrane glycoprotein; coagulation and angiogenesis (F3 gene)	Upregulated (microenvironment-associated)	Poor (progression, metastasis)
CEACAM5 (45, 46)	Cell surface glycoprotein; adhesion and signaling (CEA gene)	~50 (in lymph nodes/NSCLC)	Poor (micrometastasis, recurrence)
MUC1 (47, 48)	Transmembrane mucin; aberrant glycosylation for signaling/resistance (MUC1 gene)	~70 (high in resistant subtypes)	Poor (osimertinib resistance, short OS)
Nectin-4 (32, 49)	Cell adhesion molecule; promotes invasion (NECTIN4 gene)	~60 (membrane/cytoplasmic)	Poor (invasion, metastasis)

*Expression Rate*: Approximate percentages derived from immunohistochemistry (IHC) or next-generation sequencing (NGS) data; primarily NSCLC unless specified. *Prognostic Association*: “Poor” indicates adverse outcomes (shorter PFS/OS). (RTK, Receptor Tyrosine Kinase; MAPK, Mitogen-Activated Protein Kinase; PI3K, Phosphoinositide 3; ERBB2, Erythroblastic Leukemia Viral Oncogene Homolog 2; NSCLC, Non-Small Cell Lung Cancer; PFS, Progression-Free Survival; OS, Overall Survival; EGFR, Epidermal Growth Factor Receptor; MET, Mesenchymal Epithelial Transition; TACSTD2, Tumor-Associated Calcium Signal Transducer 2; HGF, Hepatocyte Growth Factor; TKI, Tyrosine Kinase Inhibitor; B7-H3, B7 Homolog 3; SCLC, Small Cell Lung Cancer; PD-L1, Programmed Death-Ligand 1; VTCN1, V-Set Domain Containing T Cell Activation Inhibitor 1; FOLR1, Folate Receptor 1; FRα, Folate Receptor Alpha; F3, Coagulation Factor III; TF, Tissue Factor; CEA, Carcinoembryonic Antigen; MUC1, Mucin 1; NECTIN4, Nectin Cell Adhesion Molecule 4; IHC, Immunohistochemistry; NGS, Next-Generation Sequencing.

The linker serves a critical determinant of ADC therapeutic efficacy and safety by governing payload release kinetics. Based on cleavage mechanisms, linkers are broadly categorized into cleavable types linkers—including enzyme-cleavable linkers, acid-sensitive linkers, redox-sensitive linkers, and self-immolative linkers—which account for 80-90% of approved ADCs, and non-cleavable types (51, 52). Enzyme-cleavable linkers achieve high tumor-specific payload release (>80%) and generate substantial bystander effects; however, their clinical application is limited by premature systemic cleavage and heterogeneous protease expression across tumors (53–55). Acid-sensitive linkers designed to be cleaved in the acidic lysosomes (pH 4.5-6.5) of tumor cells, leverage this condition but demonstrate compromised plasma stability, resulting in suboptimal drug delivery (53, 56). Redox-sensitive linkers face a fundamental clinical constraint due to the inter- and intra-tumoral heterogeneity of glutathione concentrations, leads to unpredictable and often insufficient payload release, which severely limits their reliability across patient populations (51, 53). Self-immolative linkers effectively maintain payload integrity but introduce additional complexity, as their efficiency depends entirely on the initial cleavage event, potentially creating bottlenecks in drug release kinetics (57, 58). Non-cleavable linkers exhibit exceptional plasma stability (>200-hour half-life) and minimal off-target toxicity, establishing a favorable safety profile. However, this stability is accompanied by therapeutic limitations such as low release efficiency (20-30%) and absence of bystander effects, which are particularly problematic in heterogeneous malignancies (**Table 2**) (23, 51, 54, 56, 59).

**Table 2:** Classification and characteristics of ADC linkers.

Linker type	Subtype	Cleavage mechanism	Example ADC/Payload
Cleavable	Enzyme-cleavable	Hydrolysis by tumor/lysosomal enzymes via peptide bonds	mc-Val-Cit-PABC-MMAE (Brentuximab vedotin) (51, 53); GGFG-DXd (Datopotamab deruxtecan) (58).
	pH-sensitive	Protonation in acidic TME/lysosomes (pH 4.5-6.5)	Hydrazone-DOX (BR96-DOX) (51); Silyl ether-MMAE (53).
	Redox-sensitive	Reduction of disulfide bonds by high glutathione (GSH) in tumor cells.	Disulfide-DM4 (IMGN901) (51); SPDB-DM4 (53).
	Self-immolative	Spacer undergoes 1, 4/1, 6-elimination or cyclization post-cleavage to release native payload.	PABC-MMAE (Polatuzumab vedotin) (57); PABC-DXd (Enhertu) (58).
Non-cleavable	Non-cleavable	No specific cleavage; relies on lysosomal degradation of entire ADC.	SMCC-DM1 (Kadcyla) (51, 56); Maleimide-MC (53).

Val-Cit, Valine-Citrulline; PABC, p-aminobenzyloxycarbonyl; MMAE, monomethyl auristatin E; GGFG, Gly-Gly-Phe-Gly; DXd, deruxtecan; TME, tumor microenvironment; DOX, doxorubicin; GSH, glutathione; SMCC, succinimidyl-4-(N-maleimidomethyl) cyclohexane-1-carboxylate.

**Figure 1:**
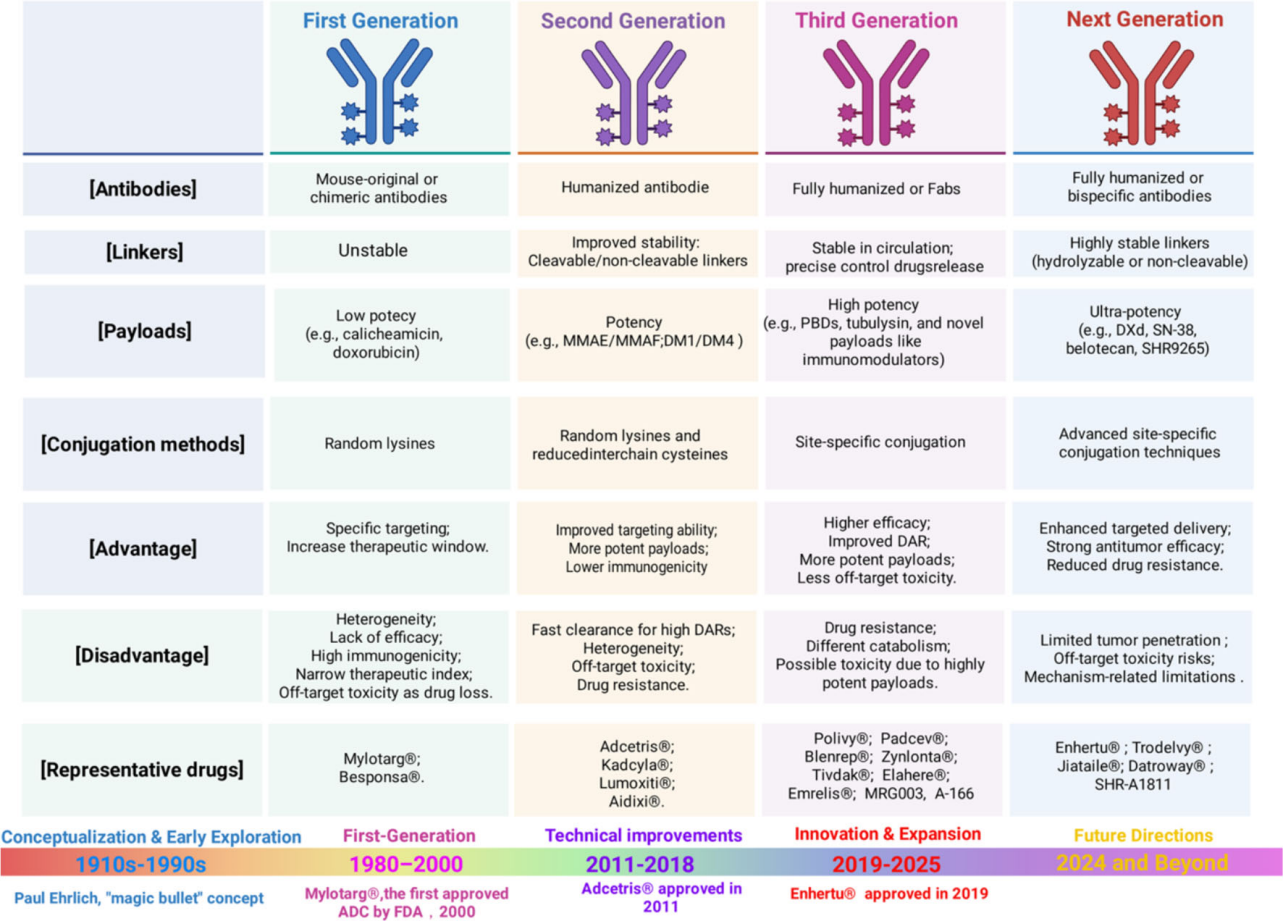
Evolution of ADCs across generations. This figure summarizes the key features of first-, second-, third-, and next-generation ADCs, comparing antibody formats, linker stability, payload potency, and conjugation strategies. Representative drugs, along with major advantages and limitations of each generation, are highlighted, illustrating the technological progression toward improved stability, efficacy, safety, and targeted antitumor activity. Image created with Biorender.com, with permission.

Payloads represent the core therapeutic components of ADCs, executing antitumor activity through direct cytotoxic activity on target cells. Early-generation ADCs predominantly employed DNA damaging agents (e.g., doxorubicin, calicheamicin and duocarmycin) as payloads, conjugated to murine-derived antibodies or humanized IgG4 monoclonal antibodies through non-cleavable linkers, the insufficient linker stability frequently led to premature payload release, resulting in systemic exposure and marked off-target toxicity (14, 17, 18). In contrast, second-generation ADCs (e.g., Brentuximab vedotin, Trastuzumab emtansine(T-DM1)) are defined by use of microtubule inhibitors as payloads, including maytansinoids (DM1, DM4) and auristatins (monomethyl auristatin E (MMAE) and monomethyl auristatin F(MMAF)), Due to their significant systemic and dose-limiting toxicities (e.g., myelosuppression, peripheral neuropathy), which force the maximum tolerated dose near the minimum effective dose, these ADCs payloads exhibit a narrow therapeutic index (or window) and consequently have limited efficacy against tumors with low antigen expression (16, 60–65). The third-generation ADCs predominantly employ topoisomerase I inhibitors (such as Deruxtecan(DXd), and SN-38) and DNA alkylating agents (e.g., pyrrolobenzodiazepine dimers(PBD)) as payloads, which exhibit an improved therapeutic window for solid tumors (66, 67). Representative agents including Trastuzumab Deruxtecan (T-DXd) and Sacituzumab Govitecan (SG) have demonstrated remarkable efficacy in advanced NSCLC (28, 68–73). Despite their enhanced efficacy, some unique toxic reactions such as interstitial lung disease (ILD) continue to limit the further application in NSCLC (**Table 3**) (79, 83, 84). In recent years, research focus has gradually shifted toward novel payload systems, including metal complexes, naturally derived toxins, protein degraders, and immunomodulatory agents (85–88)(**Figure 1**). These next-generation payloads are designed to overcome the toxicity limitations of conventional chemotherapeutic drugs while offering improved tissue penetration and immunomodulatory capabilities, thereby opening new avenues for ADCs clinical applications in NSCLC.

**Table 3:** Summary of antibody–drug conjugates approved worldwide for clinical use, as of November 2025.

Drugs	Trade names (Company)	Approved date	Approved countries	Target antigens	Payloads	Linkers	DAR	Approved indications
Gemtuzumab ozogamicin	Mylotarg^®^ (Pfizer)	2000/5/17; 2017/9/1 (Reapproved)	FDA/EMA/PMDA	CD33	N-acetyl-γ-calicheamicin	hydrazone	2-3	newly diagnosed and R/R CD33+ AML.
Brentuximab vedotin (62)	Adcetris^®^(Seagen/Takeda)	2011/8/19	FDA/EMA/PMDA/NMPA	CD30	MMAE	mc-VC-PABC	4	R/R HL and sALCL; peripheral T-cell lymphoma; DLBCL.
Trastuzumab emtansine (74)	Kadcyla^®^ (Roche)	2013/2/22	FDA/EMA/PMDA/NMPA	HER2	DM1	SMCC	3.5	HER2+ metastatic breast cancer.
Inotuzumab ozogamicin (72)	Besponsa^®^ (Pfizer)	2017/6/28	FDA/EMA/PMDA	CD 22	N-acetyl-γ- calicheamicin	hydrazone	2-8	adults with R/R B-cell precursor ALL;pediatric patients with R/R ALL.
Moxetumomab pasudotox-tdfk (75)	Lumoxiti^®^ (AstraZeneca)	2018/9/13; 2021withdrew	FDA/EMA	CD22	PE38	mc-VC-PABC	NA	R/R hairy cell leukemia after systemic therapies.
Polatuzumab vedotin (76)	Polivy^®^ (Roche)	2019/6/10	FDA/EMA	CD79b	MMAE	mc-VC-PABC	3-4	R/R DLBCL.
Enfortumab vedotin (64)	Padcev^®^ (Astellas/Seagen)	2019/12/18	FDA	Nectin-4	MMAE	mc-VC-PABC	3.8	La/M urothelial carcinoma; advanced bladder cancer.
Trastuzumab deruxtecan (77)	Enhertu^®^ (Daiichi Sankyo)	2019/12/20	FDA/EMA/PMDA	HER2	Dxd	tetrapeptide	7-8	HER2+/HER2-low metastatic breast cancer; HER2+ advanced gastric cancer/solid tumors; HER2-mutant metastatic NSCLC.
Sacituzumab govitecan (67)	Trodelvy^®^ (Gilead)	2020/4/22	FDA	TROP2	SN-38	CL2A	7-8	metastatic TNBC and HR+/HER2- metastatic breast cancer received systemic therapies
Belantamab mafodotin (60)	Blenrep^®^ (GSK)	2020/8/5; 2022, withdrew	FDA/EMA	BCMA	MMAF	mc	4	R/R multiple myeloma received prior therapies.
Cetuximab sarotalocan (78)	Akalux^®^ (Rakuten Medical)	2020/9/25	PMDA	EGFR	IRDye700DX	NA	1.3-3.8	unresectable head and neck cancer.
Loncastuximab tesirine (55)	Zynlonta^®^ (ADC Therapeutics)	2021/4/23	FDA	CD19	PBD-dimer (SG3199)	dipeptide	2.3	R/R large B-cell lymphoma after systemic therapy.
Disitamab vedotin (79)	Aidixi^®^ (RemeGen)	2021/6/8	NMPA	HER2	MMAE	mc-VC-PABC	4	HER2-overexpressing (IHC 2+/3+) l La/M gastric cancer or La/M urothelial carcinoma.
Tisotumab vedotin (65)	Tivdak^®^ (Seagen)	2021/9/20	FDA	TF	MMAE	mc-VC-PABC	4	recurrent or metastatic cervical cancer.
Mirvetuximab soravtansine (63)	Elahere^®^ (ImmunoGen)	2022/11/14	FDA	FRα	DM4	Sulfo-SPDB	3	epithelial ovarian, fallopian tube, or peritoneal cancer after systemic treatments.
Sacituzumab tirumotecan (80)	Jiataile^®^ (Kelun-Biotech)	2024/6/10	NMPA	Trop-2	Belotecan-derivative	MsPr	7.4	La/M TNBC; EGFR-mutant locally advanced or metastatic NSCLC.
Datopotamab Deruxtecan (73)	Datroway^®^ (AstraZeneca/Daiichi Sankyo)	2024/12/27	FDA	Trop-2	Dxd	deruxtecan	4-8	unresectable or recurrent/metastatic HR+/HER2- breast cancer after chemotherapy.
Telisotuzumab vedotin (61)	Emrelis^®^ (AbbVie Inc.)	2025/5/14	FDA	c-Met	MMAE	mc-val-cit-PABC	2-4	La/M non-squamous NSCLC after systemic therapy.
Trastuzumab rezetecan (81)	SHR-A1811 (Jiangsu Hengrui)	2025/5/29	NMPA	HER2	SHR9265	mc-vc-PABC	6	unresectable La/M NSCLC received at least one systemic therapy.
Trastuzumab botidotin (82)	A-166 (Kelun Biotech)	2025/10/17	NMPA	HER2	AS269	Val-Cit	2	unresectable or metastatic HER2+ breast cancer received anti-HER2 therapies.
Becotatug vedotin (82)	MRG003 (Lepu Biopharma)	2025/10/30	NMPA	EGFR	MMAE	Val-Cit	3.8	recurrent or metastatic nasopharyngeal carcinoma after at least two prior lines of systemic therapy.

*DAR*, Drug-antibody ratio; *FDA*, US Food and Drug Administration; *EMA*, European Medicines Agency; *PMDA*, Pharmaceuticals and Medical Devices Agency of Japan; *R/R*, relapsed or refractory; *AML*, Acute myeloid leukemia; *NMPA*, National Medical Products Administration of China; *MMAE*, Monomethyl auristatin E; *mc-VC-PABC*, maleimidocaproyl-valine-citrulline-p-aminobenzoyloxycarbonyl; *HL*, Hodgkin lymphoma; *sALCL*, systemic anaplastic large cell lymphoma; *DLBCL*, Diffuse large B-cell lymphoma; *mc*, maleimidocaproyl; *HER2*, human epidermal growth factor receptor 2; *DM1*, derivative of maytansine 1; *SMCC*, Succinimidyl trans-4-(maleimidylmethyl) cyclohexane-1-carboxylate; *HER2+*, HER2-positive; *ALL*, Acute lymphoblastic leukemia; *PE38*, a 38kD fragment of Pseudomonas exotoxin A; *PABC*, peptide-mc linker; *Nectin-4*, Nectin cell adhesion molecule-4; *DXd*, Exatecan derivative for ADC; *NSCLC*, Non-small cell lung cancer; *TROP2*, Trophoblast cell surface antigen 2; *MsPr*, methyl sulfonyl pyrimidine; *SN3*, active metabolite of irinotecan; *CL2A*, a cleavable complicated; *La/M*, locally advanced or metastatic*; TNBC*, Triple- negative breast cancer; *BCMA*, B-cell maturation antigen; *MMAF*, monomethyl auristatin-F; *EGFR*, Epidermal growth factor receptor; *SG3199(PBD)*, Pyrrolobenzodiazepine, *PBD* pyrrolobenzodiazepine; *TF*, Tissue factor; *FRα*, Folate receptor alpha; *DM4*, ravtansine; *Sulfo-SPDB*, N-Succinimidyl 4-(2-pyridyldithio)-2-sulfobutanoate; *c-MET*, cellular-Mesenchymal to Epithelial Transition factor; *SHR9265*, exatecan derivative, *AS269AS269* exatecan-based topoisomerase I inhibitor; *Val-Cit*, valine-citrulline.

**Figure 2:**
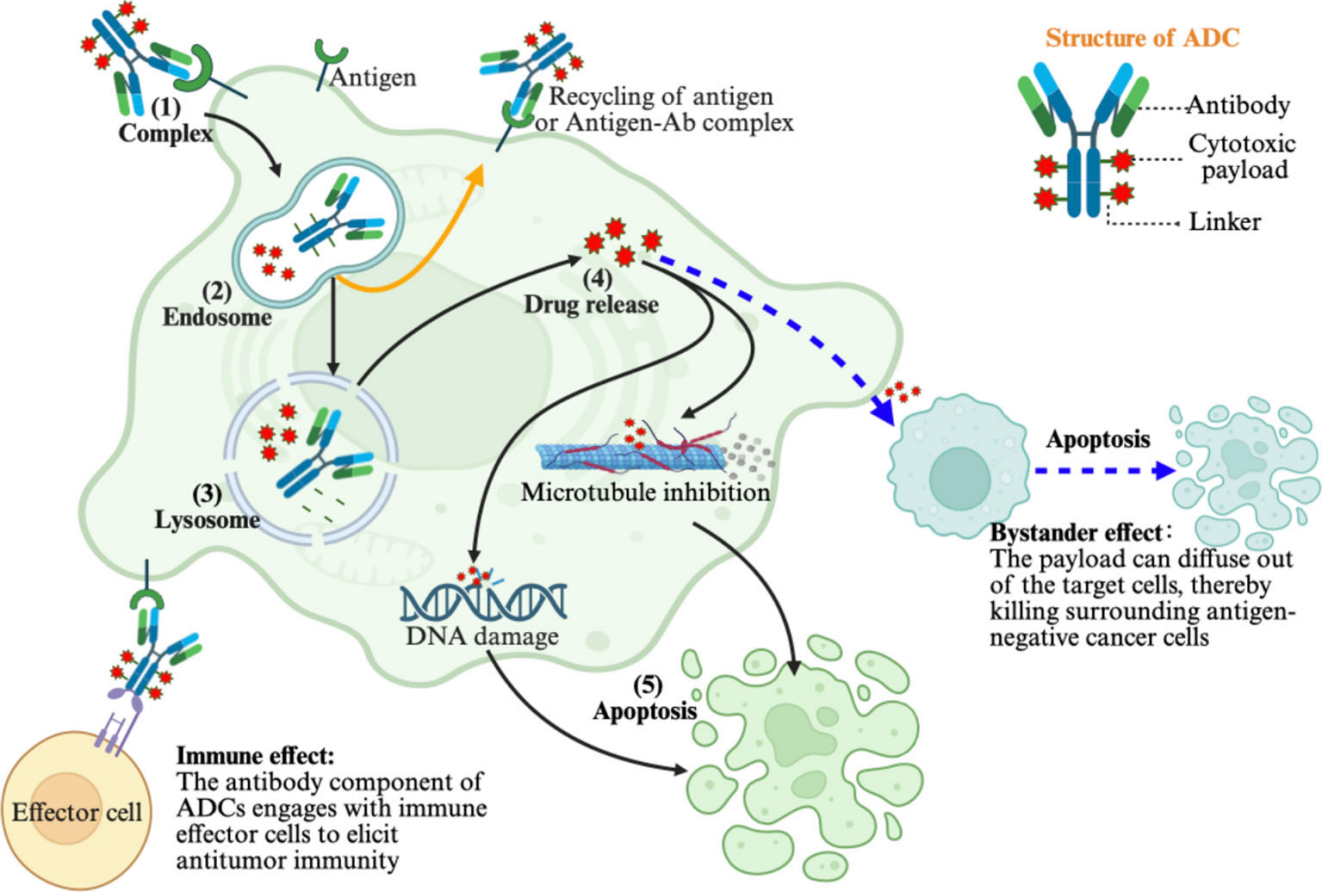
Schematically illustrates the multifaceted antitumor mechanisms of ADCs. The primary pathway involves ADC binding to its cognate target antigen on the tumor cell surface, initiating receptor-mediated endocytosis and subsequent lysosomal trafficking. Lysosomal degradation facilitates the selective release of the potent cytotoxic payload, which induces programmed cell death via mechanisms including DNA damage or microtubule disruption. Moreover, the membrane-permeable nature of certain payloads enables a critical bystander effect, whereby the cytotoxic agent diffuses from antigen-positive target cells into neighboring antigen-negative malignant cells, thereby overcoming tumor heterogeneity and expanding the therapeutic reach. In addition, certain ADCs retain the Fc function of the antibody, enabling them to eliminate tumor cells through mechanisms such as antibody-dependent cellular cytotoxicity (ADCC) or antibody-dependent cellular phagocytosis (ADCP) upon binding to tumor antigens. Image created with Biorender.com, with permission.

The antitumor mechanisms of ADCs are multifaceted and can be briefly summarized as follows: internalization pathway, bystander effect, and immune effect (**Figure 2**) (23, 59, 89). The classical internalization pathway begins with antigen binding and cellular uptake of the ADC, followed by lysosomal degradation and payload release. The released cytotoxin then induces tumor cell apoptosis or necrosis through mechanisms such as cytoskeletal disruption, genomic integrity compromise, or transcriptional interference (89, 90). Beyond this primary pathway, membrane-permeable payloads may also elicit a bystander effect, in which cytotoxic agents diffuse into neighboring, thereby extending therapeutic efficacy in heterogeneous tumors with negative or low antigen expression (24, 91). This mechanism is particularly relevant in heterogeneous tumors, where negative or low antigen expression limits conventional ADC efficacy, and the bystander effect enhances overall therapeutic activity (92). Moreover, some ADCs retain the Fc-mediated effector functions of the antibody, allowing them to mediate the elimination of tumor cells via antibody-dependent cellular cytotoxicity (ADCC) or antibody-dependent cellular phagocytosis (ADCP) following antigen engagement, promoting the release of tumor-associated antigens and danger signals, activates antitumor immune responses, and provides a rationale for combinatorial strategies with immune checkpoint inhibitors or other immunotherapies to achieve synergistic tumor-killing effects (25, 84, 92).

## 3 Clinical trials in advanced NSCLC

### 3.1 HER2-targeted ADCs

HER2, a member of the pan-HER receptor tyrosine kinase family, serves as a pivotal oncogenic driver in NSCLC. HER2 activation in NSCLC encompass three primary mechanisms: activating mutations (incidence: 1-6.7%), gene amplifications (1.4-22%), and protein overexpression (6-35%), with mutation frequencies notably elevated in Chinese populations (93, 94). HER2 activation promotes oncogenic signaling networks that promote tumor proliferation, survival, invasion, and epithelial-mesenchymal transition, correlating with aggressive disease progression, enhanced metastatic potential, resistance to EGFR tyrosine kinase inhibitors (EGFR-TKIs), and unfavorable clinical outcomes (10, 28, 70, 95).

Multiple ADCs targeting HER2 have demonstrated clinically meaningful antitumor activity in HER2-positive NSCLC. Trastuzumab emtansine (T-DM1), which conjugates anti-HER2 monoclonal antibody trastuzumab to the microtubule inhibitor DM1, induces HER2 internalization and lysosomal degradation, resulting in targeted intracellular payload release (96–98). The phase II study(Clinical trial number:JapicCTI-194620) (99) enrolled 22 patients with HER2 exon 20 insertion−mutant NSCLC who had received 1–2 prior lines of chemotherapy and administered T−DM1 (3.6 mg/kg every 21 days), resulting in an objective response rate(ORR) of 38.1% (90% CI: 23.0–55.9%), a median duration of response(mDOR) of 3.5 months, median progression−free survival(mPFS) of 2.8 months, and median overall survival(mOS) of 8.1 months. In the phase II study (96) of trastuzumab emtansine (T-DM1) for 49 pretreated patients with advanced HER2-overexpressing NSCLC (29 IHC 2+ and 20 IHC 3+), an ORR of 20% (consisting of 4 partial responses lasting 2.9 to 10.8 months) was confined to the IHC 3+ cohort, with no significant survival difference between cohorts.

Trastuzumab deruxtecan (T-DXd) is composed of a humanized anti-HER2 IgG1 antibody, a cleavable tetrapeptide-based linker, and topoisomerase I inhibitor payload (100). Early phase I study investigations demonstrated substantial activity, with an ORR of 72.7% and mPFS of 11.3 months in a cohort of 11 patients with HER2-mutant NSCLC (101). The phase II DESTINY-Lung01 trial (a single-arm, phase 2 trial, NCT03505710) (N = 91) established an ORR of 55% with mPFS and overall survival (OS) of 8.2 and 18.6 months, respectively (70, 102, 103). DESTINY-Lung02(phase II trial, NCT04644237) further confirmed dose-dependent efficacy: ORRs were 49.0% (5.4 mg/kg, N = 102) and 56.0% (6.4 mg/kg, N = 50), with corresponding mPFS of 9.9 and 15.4 months, however, the higher dose was associated with increased toxicities—notably ILD—leading to the establishment of 5.4 mg/kg as the recommended standard dose in clinical practice. The 5.4 mg/kg dose exhibited a more favorable safety profile while maintaining substantial intracranial activity (intracranial ORR 25.0%) (104). These findings underscore the critical role of dose optimization in balancing the remarkable efficacy of T-DXd against its dose-limiting toxicities, particularly ILD. The 5.4 mg/kg regimen represents a refined risk-benefit profile for this patient population. In the Chinese subset analyzed in DESTINY-Lung05(NCT05610686) (N = 72), the ORR reached 58.3%, with a 12-month PFS rate of 55.1% (93).

Trastuzumab rezetecan (SHR-A1811) received approval from the National Medical Products Administration (NMPA) of China in May 2025 for the treatment of previously-treated, HER2-mutant, unresectable locally advanced or metastatic NSCLC (81). In the HORIZON-Lung study(phase 2, single-arm study, NCT04818333) (N = 94), an ORR of 73% was observed; however, hematologic toxicities were frequently reported, including grade 3–4 neutropenia (40%), leukopenia (27%), and thrombocytopenia (11%) (105, 106).

Disitamab vedotin (RC48), already approved in China for HER2-overexpressing gastric cancer, shows expanding clinical applications (79). Among 22 patients with HER2-positive NSCLC, the overall ORR was 45.5% with mPFS of 7.5 months. Subgroup analyses revealed differential efficacy across treatment regimens: RC48 monotherapy yielded an ORR of 47.7% and mPFS of 8.1 months; combination with HER2-TKIs resulted in an ORR of 50.0%; whereas the triplet regimen of RC48 plus platinum-based chemotherapy and bevacizumab achieved a markedly higher ORR of 71.4% (107).

The phase II trial represents the first report on the efficacy and safety of the bispecific HER2-directed antibody-drug conjugate TQB2102 in the neoadjuvant setting for HER2-positive breast cancer, demonstrating both robust antitumor activity and a manageable safety profile (108). Separately, preclinical studies support the antitumor potential of other HER2-targeted ADCs— including A166, and ARX788—in HER2-positive models, though clinical validation in advanced NSCLC remains under investigation (101, 109–115).

### 3.2 TROP2-targeted ADCs

TROP2, a transmembrane glycoprotein involved in the regulation of cell proliferation, is broadly expressed throughout the respiratory epithelium (116). Its overexpression is correlated with elevated cancer-specific mortality in lung adenocarcinoma, mediated through enhanced proliferative signaling, increased invasive capacity, metastatic competence, and resistance to immunotherapy (117–120). Currently, three TROP2-targeting ADCs—Datopotamab deruxtecan (Dato-DXd), Sacituzumab ovitecan (SG), and Sacituzumab tirumotecan (Sac-TMT)—are under clinical evaluation or use in NSCLC.

Dato-DXd consists of a humanized anti-TROP2 IgG1 monoclonal antibody conjugated to Deruxtecan, a potent topoisomerase I inhibitor, via a plasma stable, tetrapeptide-based linker. This design enables targeted payload release upon lysosomal proteolytic cleavage, minimizing systemic exposure and off-target toxicity (120). In the TROPION-PanTumor 01 trial(NCT03401385) (N = 210), patients with unresectable advanced/metastatic NSCLC treated with Dato-DXd achieved an ORR of 26%, with a median duration of response (mDOR) of 10.5 months, mPFS of 6.9 months, and mOS of 11.4 months (116). The TROPION-PanTumor 02 study(NCT05463060), a Phase 1/2 trial in Chinese patients with advanced solid tumors, reported an ORR of 45.0% and a disease control rate (DCR) of 85.0% in the NSCLC cohort (n=40), with mDOR of 8.3 months (121, 122). In the TROPION-LUNG01 trial (NCT04656652), Dato-DXd demonstrated a numerical but non-significant improvement in mPFS compared with docetaxel (4.4 vs. 3.7 months; HR = 0.89) in patients with previously treated advanced NSCLC (123). The TROPION-LUNG05 study(NCT04484142) (N = 137) further reported an ORR of 35.8%, mDOR of 7.0 months, and disease control rate (DCR) of 78.8%, with consistent responses observed in the EGFR-mutant subgroup (ORR 34%) (124, 125).

Sacituzumab tirumotecan (Sac-TMT, also known as MK-2870/SKB264) is a novel TROP2-targeting ADC that, following internalization, releases a topoisomerase I inhibitor payload, resulting in DNA damage, cell cycle arrest, and apoptosis (126). Across multiple clinical trials, Sac-TMT has demonstrated consistent antitumor activity. In the phase 1/2 KL264–01 trial (NCT04152499), Sac-TMT demonstrated an ORR of 40% (17/43) and a mPFS of 6.2 months in 43 previously treated advanced NSCLC patients (34). In the phase 2 SKB264-II-08 trial (NCT05631262, N = 64) involving previously treated EGFR-mutant NSCLC patients, achieved an ORR of 34% (22/64) and a mPFS of 9.3 months (34). In the phase 3 OptiTROP-Lung04 trial(NCT05870319) (n=376) in EGFR-mutated NSCLC after EGFR-TKI failure, sac-TMT significantly improved mPFS (8.3 vs 4.3 months) and 18-month OS rate (65.8% vs 48.0%) compared to platinum-chemotherapy (80). In a head-to-head comparison with docetaxel for EGFR-mutant NSCLC, Sac-TMT demonstrated significant superiority across all efficacy endpoints: ORR (45% vs 16%), mPFS (6.9 vs 2.8 months), and 12-month OS rates (73% vs 54%)(NCT05631262) (127). These data support Sac-TMT as a promising therapeutic option, particularly for EGFR-mutant NSCLC.

Sacituzumab govitecan (SG, IMMU-132) employs a hydrolysable linker to conjugate the anti-TROP2 antibody with SN-38, the active metabolite of irinotecan, enabling localized drug release both intracellularly and within the tumor microenvironment (67, 128). The membrane-permeability of SN-38 also facilitates a potent bystander effect, broadening its applicability across heterogeneous tumors (129, 130). The open-label, phase III EVOKE-01(NCT05089734), which compared SG(n=299) to docetaxel(n=304) in previously treated advanced NSCLC, SG showed numerical but non-significant improvements in mOS (11.1 vs. 9.8 months) and mPFS (4.1 vs. 3.9 months) (131). Although the primary endpoint was not met, SG exhibited a trend toward survival benefit and a more favorable tolerability profile. In the phase 2 TROPiCS-03 study(NCT03964727) evaluating SG as second-line therapy in 43 extensive-stage SCLC (ES-SCLC) patients, the ORR was 41.9% among 43 evaluable patients, with a median duration of response of 4.73 months, mPFS of 4.40 months, and mOS of 13.60 months (132). Several other TROP2-targeting ADCs, such as SHR-A1921, BL-M02D1, and LCB84 are ongoing clinical trials for NSCLC (133, 134), and the research results are worthy of continuous attention.

### 3.3 c-MET-targeted ADCs

c-MET, also known as hepatocyte growth factor receptor (HGFR), is overexpressed frequently in solid tumors, including NSCLC, rendering it a promising target for the development of anticancer therapeutics (35, 135–137). Telisotuzumab vedotin (Teliso-V; ABBV-399), the first c-MET-targeting ADC approved for advanced NSCLC, comprises a humanized anti-c-MET mAb (telisotuzumab) site-specifically conjugated to the potent microtubule inhibitor MMAE via a protease-cleavable valine-citrulline dipeptide linker (61, 138, 139). Consistent antitumor activity of Teliso-V has been demonstrated across multiple clinical trials in NSCLC. In a phase I/II study (NCT02099058) evaluating Teliso-V combined with erlotinib in 42 c-MET-positive NSCLC patients, the efficacy-evaluable population (n=36) achieved an ORR of 30.6%, DCR of 86.1%, and mPFS of 5.9 months. The EGFR-mutant subgroup (n=28) showed comparable efficacy, with an ORR of 32.1%, DCR of 85.7%, and mPFS of 5.9 months (140). Another trial (NCT02099058.) (n=52) revealed that among 40 response-evaluable patients with c-Met-overexpressing NSCLC, Teliso-V monotherapy yielded an ORR of 23%, with median duration of response of 8.7 months and mPFS of 5.2 months (141). The LUMINOSITY trial (NCT03539536), which specifically enrolled patients with advanced c-MET-overexpressing NSCLC (n=172) who had received ≤2 prior lines of therapy, reported an ORR of 28.6%, a median duration of response of 8.3 months, mOS of 14.5 months, and mPFS of 5.7 months (142). These consistent response rates across heterogeneous NSCLC populations confirm the meaningful clinical activity of c-Met-directed therapy in this setting.

The therapeutic landscape for c-MET-positive solid tumors continues to expand with the emergence of numerous c-MET-targeting ADCs. Preclinical candidates currently under investigation include STI-D0602, hucMet27-based ADCs, cIRCR201-dPBD, P3D12-vc-MMAF, LAV- and HAV-ADCs, PCMC1D3-Duocarmycin SA, as well as c-Met/EGFR bispecific constructs. Meanwhile, several agents have advanced into clinical evaluation, such as SHR-A1403, TR1801-ADC, RC108, BYON3521, MYTX-011, ABBV-400, REGN5093-M114, and AZD9592, persistent and growing interest in c-MET as a therapeutic target (143–146).

### 3.4 Novel ADCs targets: HER3, CEACAM5 and B7-H3

HER3 is broadly expressed in NSCLC and has emerged as a promising therapeutic target. The HER3-targeting ADC Patritumab deruxtecan (HER3-DXd) has shown substantial efficacy in patients with EGFR-mutated NSCLC following progression on EGFR-TKI and platinum-based chemotherapy. Initial phase I U31402-A-U102 trial (NCT03260491) results showed a confirmed ORR of 41%, with mPFS of 6.4 months and mOS of 16.2 months (147). These findings of this phase II study HERTHENA-Lung01(NCT04619004) enrolled 225 patients with advanced EGFR-mutated NSCLC previously treated with EGFR-TKIs therapy and platinum-based chemotherapy, demonstrated a confirmed ORR of 29.8%, median duration of response of 6.4 months, mPFS of 5.5 months, and mOS of 11.9 months in a comparable patient population (30). The consistent therapeutic activity observed across these studies underscores the clinical potential of HER3-DXd in this treatment-resistant NSCLC subgroup.

CEACAM5, a glycosylphosphatidylinositol-anchored membrane glycoprotein frequently overexpressed in NSCLC, plays a key role in intercellular adhesion, proliferative signaling, tumor invasion, and resistance to apoptosis (148, 149). Preclinical studies have demonstrated that CEACAM5-targeted ADCs potently suppress tumor growth in CEACAM5-positive lung cancer models (50, 150, 151).

B7-H3, a member of the B7 costimulatory family, is commonly overexpressed on tumor cells and tumor-associated vascular endothelium in NSCLC and is correlated with poor prognosis (152–154). The B7-H3–targeting ADC ifinatamab deruxtecan (I-DXd), which delivers a topoisomerase I inhibitor, has shown encouraging efficacy in early clinical trials involving pretreated extensive-stage small cell lung cancer (SCLC). Treatment with I-DXd resulted in an ORR of 54.8%, a mPFS of 5.5 months, and a mOS of 11.8 months. Notably, substantial intracranial activity was observed, with a central nervous system–confirmed response rate of 37.8% (154, 155).

**Figure 3:**
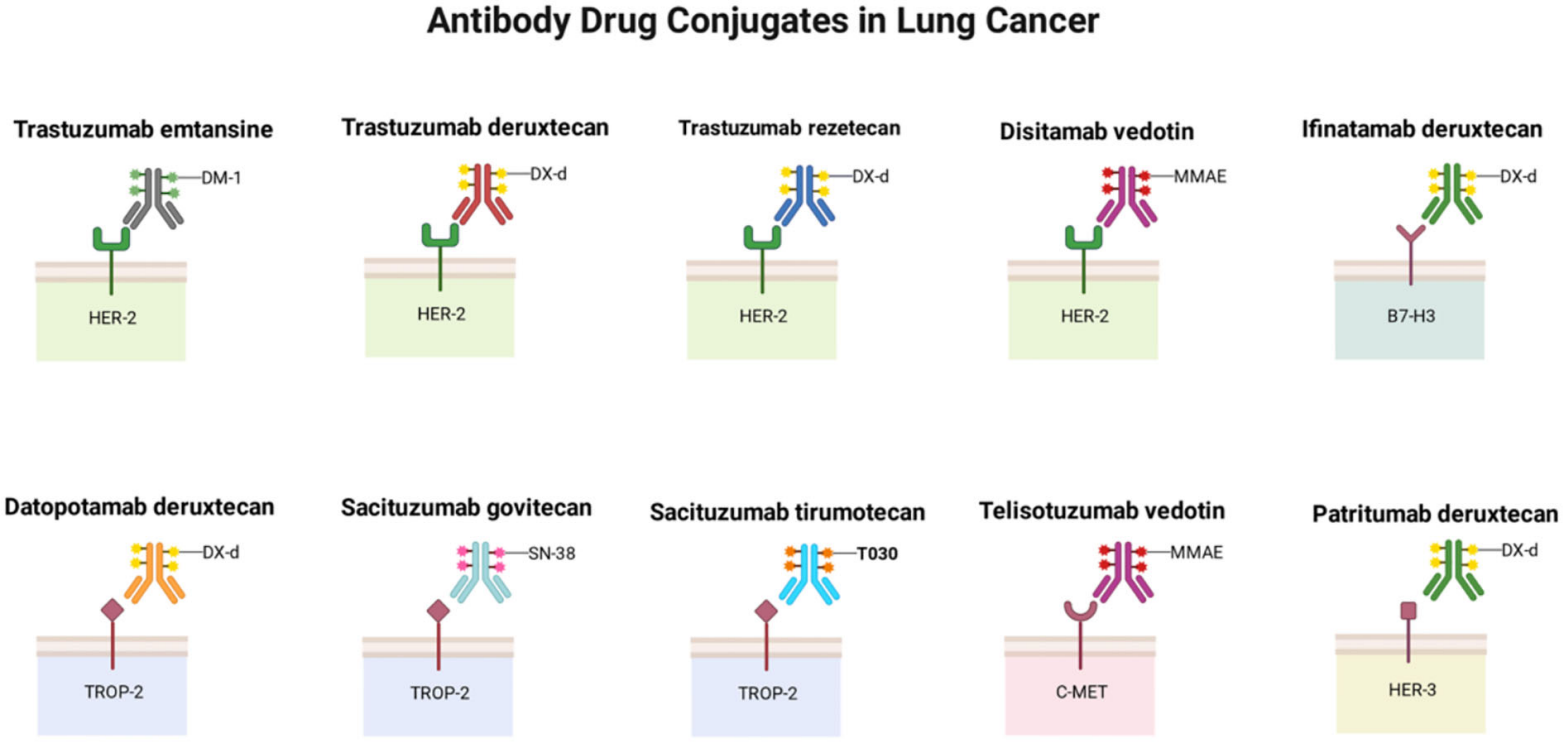
Current ADCs in lung cancer. The figure illustrates the major antibody-drug conjugates (ADCs) currently used in lung cancer treatment, which primarily include those targeting HER2, HER3, TROP−2, and c−MET for non−small cell lung cancer, as well as ADCs targeting B7−H3 for small cell lung cancer. Image created with Biorender.com, with permission.

ADCs have transformed the treatment landscape for advanced non-small cell lung cancer through their unique precision-targeted delivery mechanism. With the ongoing development of ADCs targeting a growing array of antigens—including HER2, TROP2, c-MET, HER3, CEACAM5, and B7-H3, as well as emerging ones like EGFR (to overcome resistance), DLL3 (in SCLC), FRα, and NECTIN4—this therapeutic class is increasingly positioned as a cornerstone strategy to overcome tumor heterogeneity and treatment resistance in NSCLC. (**Figure 3**).

### 3.5 Comparative analysis of ADCs architectures and clinical outcomes

The distinct clinical profiles of ADCs targeting the same antigen arise from deliberate design choices. For HER2, the superior efficacy of T-DXd (high-DAR, cleavable linker, membrane-permeable DXd payload) in HER2-mutant NSCLC is counterbalanced by its ILD risk, whereas T-DM1 (lower DAR, non-cleavable linker, DM1) shows modest activity and a different toxicity spectrum. For TROP2, Dato-DXd and sac-TMT employ different linker-topoisomerase I inhibitor systems, correlating with varied ILD rates and subtype efficacy; SG’s hydrolyzable linker and SN-38 payload underlie its pronounced hematologic and gastrointestinal toxicities. For c-MET, Teliso-V’s MMAE payload defines both its activity and neuropathy profile, while next-generation candidates (e.g., ABBV-400) are exploring alternative payloads (e.g., topoisomerase I inhibitors) to improve the therapeutic index. Thus, antibody affinity, linker stability, payload mechanism, and DAR collectively dictate an ADC’s efficacy-toxicity balance, guiding their optimized use in NSCLC.

## 4 ADC-associated adverse events

**Figure 4:**
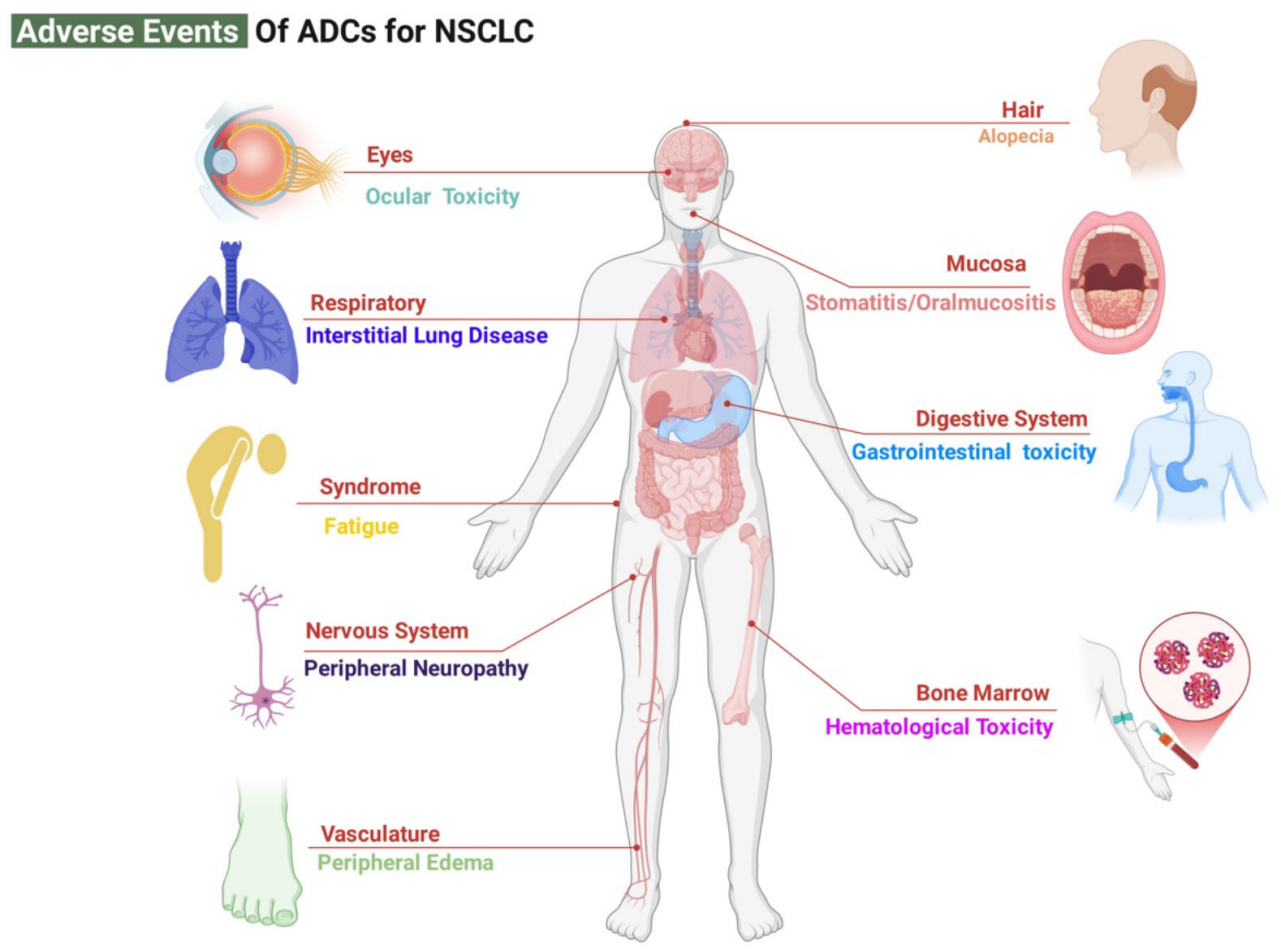
ADC-Associated Adverse Events. It summarizes the distribution of adverse events (AEs) associated with antibody-drug conjugate (ADC) therapy in non-small cell lung cancer (NSCLC), categorized by affected organ systems. The spectrum includes serious and potentially life-threatening interstitial lung disease (ILD)/pneumonitis, dose-limiting hematologic toxicities (such as neutropenia, anemia, and thrombocytopenia), and frequently occurring gastrointestinal events (including nausea and diarrhea), which collectively influence treatment tolerance and adherence. Other notable AEs encompass stomatitis, peripheral neuropathy, and ocular adverse events. Image created with Biorender.com, with permission.

Proactive prevention, vigilant monitoring, and effective management of ADC-associated toxicities are fundamental to ensuring treatment continuity and maximizing clinical benefit. The management of these adverse events is, in essence, the dynamic maintenance of the efficacy-toxicity balance. The toxicity profiles of ADCs in NSCLC are determined by the integrated physicochemical properties of their three core components—antibody, linker, and cytotoxic payload. ILD/pneumonitis, hematologic toxicities, gastrointestinal events, and other class-specific adverse effects represent the most clinically significant toxicity (**Figure 4**) (16, 156–158).

### 4.1 Interstitial lung disease/pneumonitis

ILD/pneumonitis is a serious and potentially fatal complication associated with ADC therapy in advanced NSCLC (119, 159). The reported incidence of ILD varies considerably across studies, depending on trial design, dosing regimen, and patient baseline characteristics. T-DXd demonstrates all-grade ILD in 11–28% of patients, with ≥Grade 3 events occurring in 2–7%, including fatal (Grade 5) cases in 2% (101, 101, 104, 160). Dato-DXd demonstrates all-grade ILD incidence of 6–9%, with ≥Grade 3 events in 2–3.6% (including 0.7% Grade 5) (102, 122–125, 161–163). Trastuzumab Rezetecan exhibits all-grade ILD in 10% of patients (≥Grade 3: 5%) (81, 105, 111), while SG demonstrates 5–10% all-grade and 2–3% ≥Grade 3 incidence (67, 131, 132).HER3-DXd reports all-grade ILD in 5–29%, with ≥Grade 3 events in 1–2.4% (including 1–2.4% Grade 5) (30, 164, 165). SHR-A1811 demonstrates ≥Grade 3 ILD in 2% (including one Grade 5 case) (114) and sac-TMT shows the lowest all-grade incidence at 1–2% (34, 127).

Early recognition and prompt corticosteroid intervention are critical for ILD management. Upon radiographic or clinical suspicion, ADC therapy should be immediately suspended with concurrent exclusion of infection. Grade 1 ILD necessitates temporary drug discontinuation and short-course corticosteroids (prednisone ≥0.5 mg/kg/day), with therapy resumption only after complete resolution. For Grade ≥2 ILD, permanent drug discontinuation and immediate high-dose corticosteroids (prednisone/methylprednisolone ≥1 mg/kg/day) are mandatory, followed by gradual tapering over ≥4 weeks. Severe or progressive cases require hospitalization and multidisciplinary pulmonary consultation. Baseline and serial high-resolution computed tomography monitoring—particularly during the initial six months—is recommended for early detection and improved outcomes (166, 167).

### 4.2 Hematological toxicity

Hematologic adverse events represent another major toxicity category in advanced NSCLC, with neutropenia, anemia, and thrombocytopenia being most prevalent. Neutropenia incidence ranges from 20–79% (all-grade) and 1–58% (≥Grade 3), with the highest rates observed for trastuzumab rezetecan (55% all-grade; 40% ≥Grade 3) and SG (52–79% all-grade; 28–58% ≥Grade 3) (105, 114, 122, 124, 125, 131, 132, 160, 161, 168). Anemia occurs in 30–60% (all-grade) and 4–24% (≥Grade 3) of patients, with T-DXd showing the highest incidence (48–60% all-grade; 8–16% ≥Grade 3) (30, 114, 125, 160, 161). Leukopenia affects 32–45% (all-grade) and 4–27% (≥Grade 3), while thrombocytopenia occurs in 20–28% (all-grade) and <1–29% (≥Grade 3) of patients, with HER3-DXd demonstrating the highest severe thrombocytopenia rate (21–29%) (34, 105, 122, 124, 125, 127, 160, 161, 168).

These hematologic toxicities are generally reversible through dose modification and supportive measures. Routine complete blood count monitoring before each treatment cycle enables early detection. For Grade ≥3 neutropenia or thrombocytopenia, temporary drug interruption until recovery to Grade ≤2 is recommended, followed by dose reduction or interval extension upon reinitiation. Granulocyte colony-stimulating factor (G-CSF) prophylaxis or treatment should be considered for prolonged neutropenia. Anemia management includes transfusion support or treatment delay, while platelet transfusion is reserved for severe thrombocytopenia or bleeding risk. Most events resolve with these interventions, with permanent discontinuation rarely required (162, 166, 169, 170).

### 4.3 Gastrointestinal toxicity

Gastrointestinal adverse events are frequently observed with ADC therapy in NSCLC. Nausea represents the most common all-grade event (37–65%), with incidence highest for SG (58–65%) and T-DXd (52–62%); ≥Grade 3 nausea occurs in 1–14%, most frequently with HER3-DXd (14%). Vomiting affects 20–42% of patients (all-grade) and 0–2% (≥Grade 3). Decreased appetite occurs in 20–48% (all-grade) and 5–19% (≥Grade 3), while diarrhea demonstrates the widest incidence range (4–64% all-grade), with SG showing the highest rates (59–64% all-grade; 7–13% ≥Grade 3) (30, 105, 111, 122, 125, 160–162, 164, 168).

Gastrointestinal toxicity induced by ADCs are common dose-limiting toxicities in patients with NSCLC. Management focuses on minimizing treatment interruptions while maintaining quality of life through prophylactic measures, individualized interventions, and multidisciplinary collaboration. Primary approaches include antiemetics, fluid replacement, and antidiarrheal agents tailored to symptom severity.

### 4.4 Other common toxicities

Fatigue represents the most prevalent general toxicity, affecting 20–56% of patients across ADC classes (all-grade) and 2–12% (≥Grade 3) (162). Alopecia occurs in 18–42% of patients, exclusively Grade 1–2 (105, 111, 122, 125, 160, 161, 163). Stomatitis/oral mucositis affects 49–60% of patients (all-grade), with ≥Grade 3 events in 7–9.5%, primarily associated with Dato-DXd (30, 122, 125, 161, 163). Notable class-specific toxicities include peripheral neuropathy with Teliso-V (42–57% all-grade; 7–≥20% ≥Grade 3) (61, 140–142), ocular events with A166 (corneal epithelial disease: 30.9%; blurred vision: 18.5%) (115), peripheral edema with telisotuzumab vedotin (≥20% all-grade) (140–142), and abdominal pain with HER3-DXd (≥10% all-grade) (30).

## 5 Challenges and future perspectives

### 5.1 Current clinical challenges

Despite demonstrating remarkable efficacy in advanced NSCLC, ADC-based therapeutics face several significant challenges. First, substantial heterogeneity in the expression of key targeted antigens—such as HER2, TROP2, and HER3—across patient populations often limits clinical benefit in certain individuals (113, 171, 172). Second, the lack of standardized methodologies for biomarker assessment introduces potential biases in patient selection and stratification (24). Furthermore, suboptimal tumor penetration and blood-brain barrier crossing significantly compromise effective ADC delivery (164, 173, 174).

Resistance remains a significant challenge for ADC therapy in NSCLC, involving multi-step mechanisms spanning from target recognition to intracellular cytotoxicity. Key pathways include: antigen downregulation or spatial heterogeneity (e.g., reduced HER2/TROP2 expression), which directly impairs ADC binding (175, 176); altered internalization pathways (such as a shift toward caveolin-1-mediated uptake) and lysosomal dysfunction (e.g., SLC46A3 deficiency), which hinder payload release (177, 178); overexpression of ATP-binding cassette (ABC) transporters (e.g., ABCB1, ABCC1/2), a well-documented mechanism of payload efflux validated in multiple ADCs including T-DM1 and T-DXd (179); and linker instability leading to premature systemic cleavage, which reduces effective drug concentration at the tumor site (180). Collectively, these layered resistance pathways represent a major obstacle to durable clinical responses with ADC-based treatments in NSCLC.

**Figure 5:**
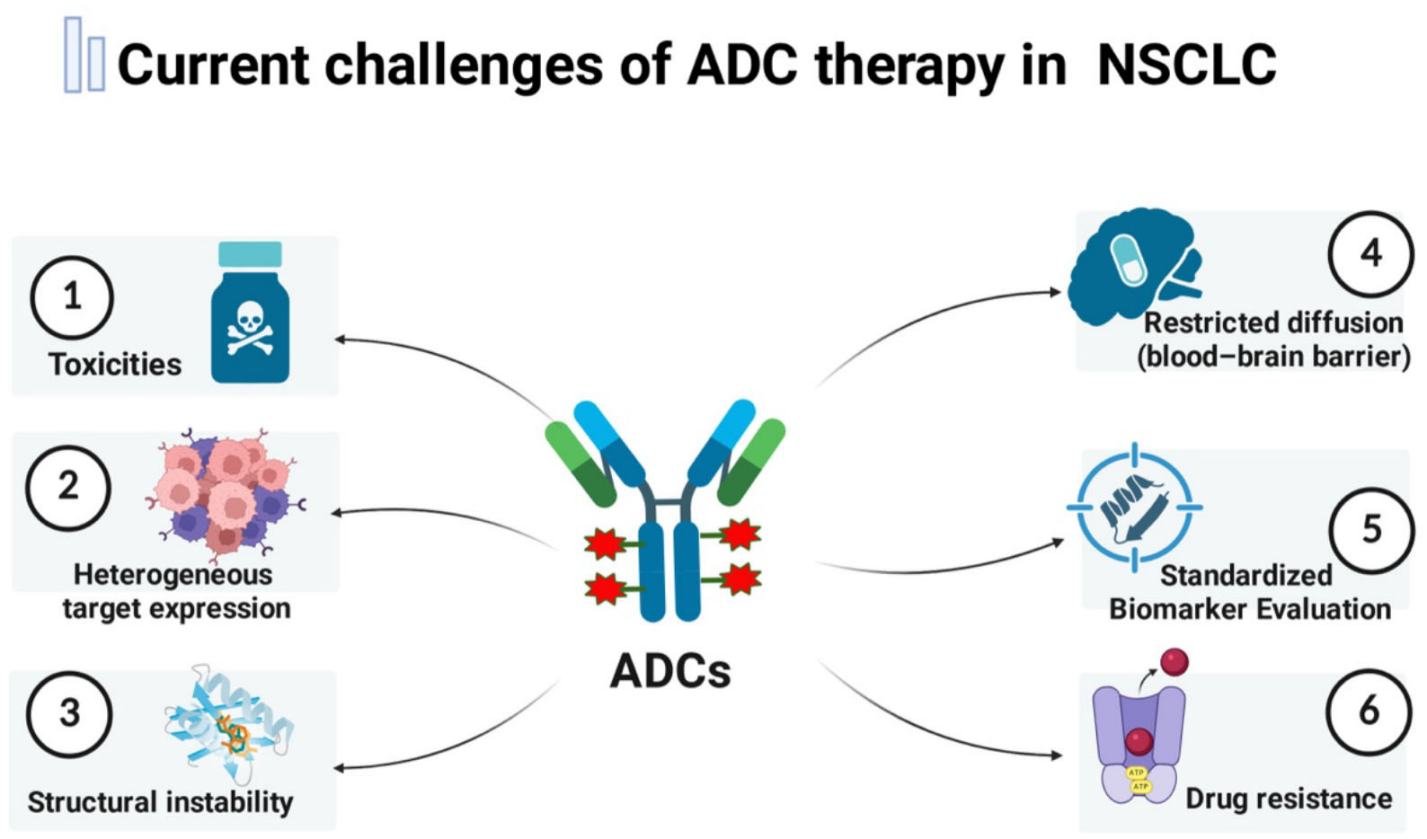
Current challenges of ADC therapy in advanced NSCLC. ADC limitations include heterogeneous target expression, variability in biomarker assessment, and poor tumor or CNS penetration. Safety issues—such as off-target toxicity, premature payload release, and ILD/pneumonitis—narrow the therapeutic window. Resistance mechanisms, including antigen loss, impaired internalization, lysosomal dysfunction, and drug efflux, further reduce efficacy. Image created with Biorender.com, with permission.

In terms of safety, despite their targeted design, ADCs still exhibit off-target effects and premature payload release, leading to toxicity profiles that partially resemble conventional chemotherapy (158). Notably, respiratory toxicities—especially ILD and pneumonitis—represent the most serious dose-limiting adverse events, with particularly high incidence observed with T-DXd and Dato-DXd (100, 101, 168). Linker instability and elevated drug-to-antibody ratios may contribute to molecular aggregation and altered pharmacokinetics, thereby narrowing the therapeutic window (15, 22, 109, 181, 182). Importantly, the frequent presence of underlying pulmonary impairment in advanced NSCLC patients exacerbates susceptibility to ILD and further constrains safe dosing parameters (24, 52). Concurrently, tumor cells employ diverse resistance mechanisms, including antigen downregulation, impaired internalization, lysosomal dysfunction, and upregulation of drug efflux pump, collectively undermining ADC therapeutic efficacy (22, 183, 184) (**Figure 5**).

Thus, a paramount clinical challenge is how to concurrently overcome therapeutic resistance and manage unique toxicities to maintain a favorable therapeutic window for ADC therapy in advanced NSCLC.

### 5.2 Future directions and strategic approaches

To systematically overcome these challenges, a multi-tiered strategic framework is essential. For optimized patient selection, developing dynamic and robust biomarker systems that integrate multi-omics profiling (26, 84, 185, 186), artificial intelligence-assisted radiomics, liquid biopsy applications, and advanced molecular imaging technologies will enable precise patient stratification and treatment tailoring (102, 187–189). In drug design, focused development of next-generation targets and innovative structural configurations—including bispecific antibodies and dual-payload ADCs—can facilitate simultaneous engagement of multiple antigens or signaling pathways, thereby better addressing tumor heterogeneity and resistance mechanisms (190, 191). Looking ahead, newly emerging next-generation ADCs strategies hold substantial promise for further enhancing therapeutic efficacy, widening the therapeutic window, and overcoming persistent limitations such as resistance, heterogeneity, and off-target toxicity. Novel linker–payload systems with superior circulatory stability and tumor-selective release, incorporating traceless or hydrophilic linkers paired with diversified payloads (e.g., topoisomerase I inhibitors, DNA alkylators, microtubule disruptors, or immunomodulatory agents) to improve intracellular delivery and bystander effects (192, 193). Optimized DAR enabled by precise site-specific conjugation technologies, producing homogeneous ADCs with balanced loading, favorable pharmacokinetics, and reduced nonspecific toxicity (194). Conditionally activatable ADCs (e.g., probody-drug conjugates or pH/protease-sensitive masked constructs) that remain inert in normal tissues but activate selectively in the tumor microenvironment, thereby minimizing on-target off-tumor toxicity (146, 192, 195). Emerging preclinical modalities such as immunostimulatory ADCs (ISACs) delivering innate immune agonists to elicit antitumor immunity, and degrader-antibody conjugates (DACs) incorporating PROTAC-like mechanisms for catalytic degradation of oncogenic proteins, providing amplified potency and resistance mitigation (195, 196). Collectively, these advancements are poised to markedly broaden the clinical applicability of ADCs across a wider range of NSCLC. Regarding therapeutic strategies, rational combination regimens incorporating immune checkpoint inhibitors or EGFR-TKIs may generate synergistic antitumor activity while delaying the emergence of resistant clones (174, 197, 198). Additionally, continuous optimization of antibody affinity, linker stability, and payload delivery efficiency remains crucial for enhancing the overall therapeutic performance of ADCs.

**Figure 6:**
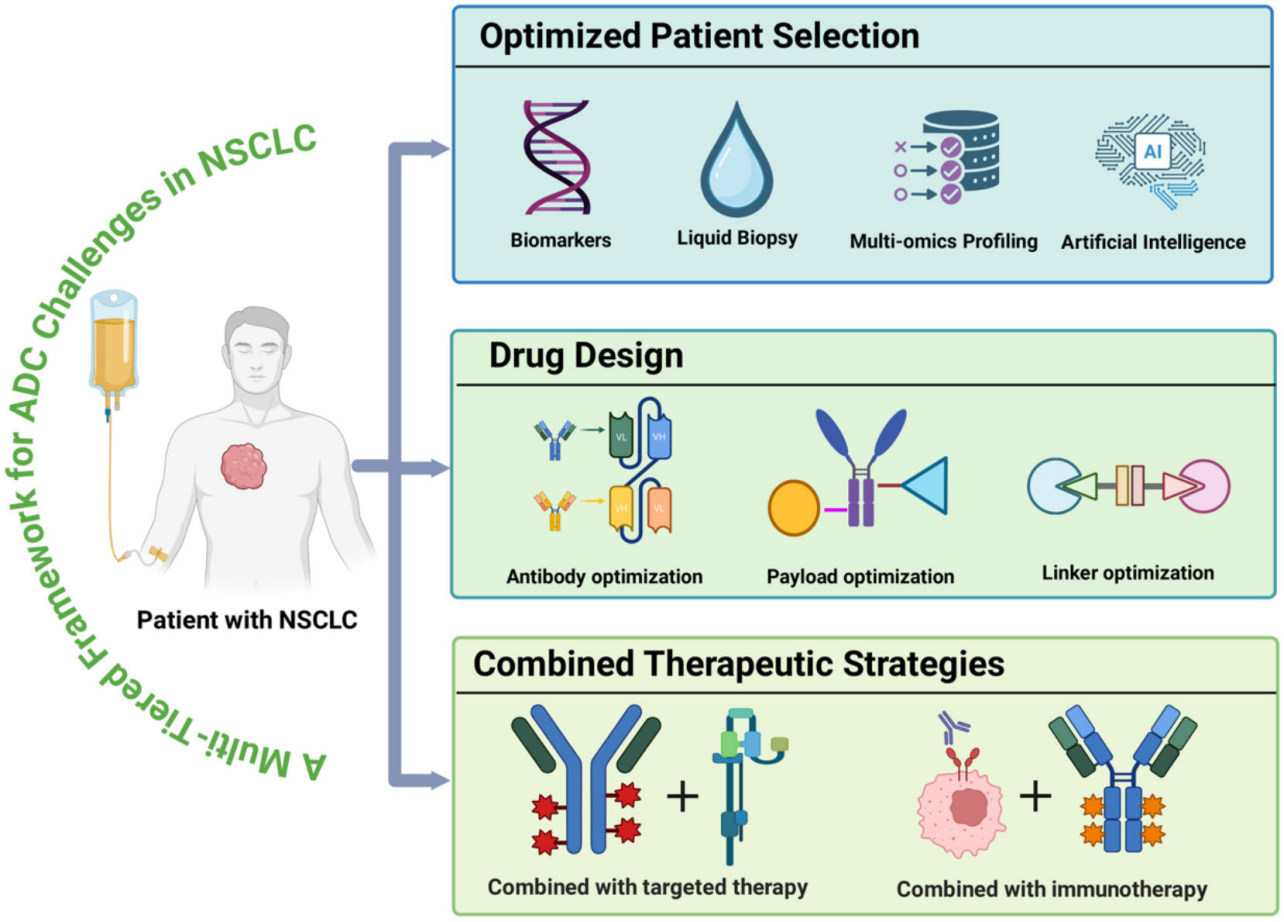
Future strategies for ADCs therapy. A multi-tiered framework outlining key directions for ADC optimization. Patient selection is enhanced through multi-omics biomarkers, AI-assisted radiomics, liquid biopsy, and advanced molecular imaging. Drug design focuses on next-generation targets, bispecific and dual-payload ADCs, and improved antibody, linker, and payload engineering. Therapeutic strategies include rational combinations such as immunotherapy or targeted-therapy to strengthen antitumor activity and delay resistance. Image created with Biorender.com, with permission.

In summary, through systematic integration of these multidimensional strategies, current limitations in ADC therapy for NSCLC can be progressively addressed, ultimately providing patients with more precise, effective, and durable treatment options (**Figure 6**).

## 6 Conclusion

ADCs have emerged as a transformative therapeutic class in advanced NSCLC, delivering clinically meaningful survival benefits through precise targeting of oncogenic drivers such as HER2, TROP2, and c-MET. This progress marks a significant milestone in the field of precision oncology, while also underscoring the critical need for rigorous monitoring standardized management protocols to optimize therapeutic index and mitigate treatment-related adverse events.

Looking forward, the continued evolution of ADCs is expected to be driven by innovations in biotechnology, including site-specific conjugation, bispecific or multi-specific antibody platforms, and the next-generation linker chemistry, all aimed at enhancing tumor selectivity, antitumor efficacy, and systemic tolerability. Rational combination strategies incorporating immunotherapy, targeted therapy, or radiotherapy are also poised to broaden the therapeutic scope of ADCs and circumvent emergent resistance mechanisms. In parallel, the integration of artificial intelligence into patient stratification, pharmacokinetic modeling, and dose optimization holds substantial potential for advancing personalized treatment paradigms.

Collectively, these developments firmly establish ADCs as cornerstone therapies in advanced NSCLC, offering renewed opportunities for prolonged survival and improved quality of life. Sustained clinical innovation and multidisciplinary collaboration will be essential to fully realize the potential of ADCs in redefining the therapeutic landscape of this challenging malignancy.
